# Safety and immunogenicity of rVSVΔG-ZEBOV-GP vaccination when dosed concurrent with mRNA COVID-19 vaccine booster doses in healthy African adults (EbolaCov): protocol for a phase IV, single-centre, single-blinded, randomised controlled trial

**DOI:** 10.1136/bmjopen-2025-102898

**Published:** 2025-09-21

**Authors:** Karishma Gokani, Alice E Taylor, Alice Packham, Jean Pierre Musabyimana, Hugor Shema, Ariane Mutabaruka, Siobhan Roche, Yemisi Takwoingi, Claudine Umuhoza, Julien Nyombayire, Claude Muvunyi, Christopher A Green

**Affiliations:** 1 University of Birmingham, Birmingham, UK; 2 Department of Infectious Diseases and Tropical Medicine, NIHR/Wellcome Clinical Research Facility, University Hospitals Birmingham NHS Foundation Trust, Birmingham, UK; 3 African Centre for Excellence in Sustainable Cooling and Cold-chain, Kigali, Rwanda; 4 Rwanda Biomedical Center, Kigali, Rwanda; 5 Public Health and Epidemiology, University of Birmingham, Birmingham, UK; 6 Center for Family Health Research, Kigali, Rwanda; 7 Rwanda Biomedical Centre, Kigali, Rwanda

**Keywords:** Disease Outbreaks, Clinical Protocols, Immunization Programs, Immunity, INFECTIOUS DISEASES, Vaccination

## Abstract

**Introduction:**

Ebola virus disease remains a significant public health concern. For protection from Ebola virus, the main target populations are epidemiologically identified and often include healthcare workers and refugees. These target populations are also routinely offered vaccines for other vaccine-preventable diseases. However, concomitant use of rVSVΔG-ZEBOV-GP with other vaccines is not recommended, given the absence of data regarding its reactogenicity and antigen-specific immunogenicity profile when co-administered. The EbolaCov trial aims to inform whether rVSVΔG-ZEBOV-GP can be administered concurrent to a Pfizer–BioNTech COVID-19 booster dose without an unacceptable increase in reactogenicity and/or loss of humoral immunogenicity to Ebola vaccine antigen.

**Methods and analysis:**

This is a single-centre, randomised, single-blinded, vaccine safety and immunogenicity study in healthy adults living in Rwanda. Seventy-two participants will be randomised in a 1:1 ratio to two study groups, the first receiving rVSVΔG-ZEBOV-GP with a placebo, the second group receiving rVSVΔG-ZEBOV-GP concurrently with a Pfizer–BioNTech COVID-19 booster dose. The primary outcome measures are quantitative serum anti-glycoprotein (GP) antibody responses, as measured by ELISA, 28 days after vaccination, and frequency and severity of adverse events in the 7 days following vaccination. Secondary outcome measures include day 28 and day 180 serum anti-GP and serum SARS-CoV-2 anti-spike protein-specific geometric mean antibody titres.

**Ethics and dissemination:**

This trial was approved by the Rwanda National Ethics Committee (reference 442/2024) and the University of Birmingham (reference ERN_2661-Jun2024). All participants were required to provide written informed consent in accordance with good clinical practice. Dissemination of results will be through conference presentations and peer-reviewed publications.

**Trial registration number:**

Pan African Clinical Trials Registry (PACTR202407764378004) and ClinicalTrials.gov (NCT06587503)

**STRENGTHS AND LIMITATIONS OF THIS STUDY**

- Safety and immunogenicity data following vaccine co-administration will be invaluable knowledge for future outbreaks when conducted in the target population and populations more representative of the settings in which they are needed.
- The study is statistically powered for non-inferior antibody responses to rVSVΔG-ZEBOV-GP 1 month after vaccination only.
- This is a single-centre study, which may limit the generalisability of the results.

## Introduction

### Ebola virus and rVSVΔG-ZEBOV-GP vaccine

 Ebola virus was first discovered in 1976, near the Ebola River in the Democratic Republic of the Congo (DRC). Of the four species belonging to the genus *Ebolavirus* that cause disease in humans, Zaire Ebola virus causes the majority of cases, including the largest Ebola outbreak to date between 2014 and 2016. The outbreak started in Guinea before spreading across to Liberia and Sierra Leone, resulting in 28 652 cases and 11 325 deaths over the subsequent 2½ years. The re-emergence of Ebola virus in Africa in 2023 highlights the need for safe, effective and sustained vaccine protection.^1^

The Ebola vaccine rVSVΔG-ZEBOV-GP was licensed by the European Medicines Agency in November 2019 and the United States Food and Drug Administration in December 2019. Countries where this vaccine has been approved for use in include Burundi, Central African Republic, the DRC, Ghana, Guinea, Rwanda, Uganda and Zambia. The Strategic Advisory Group of Experts group recommended the use of rVSVΔG-ZEBOV-GP as part of the coordinated Ebola outbreak control and more than 300 000 people in the DRC were immunised during the 2018–2020 Ebola virus outbreak.^2^ rVSVΔG-ZEBOV-GP is a live-attenuated recombinant vesicular stomatitis virus, Indiana strain, with a deletion of the VSV envelope glycoprotein (GP) and replaced with the Zaire Ebola virus Kikwit 1995 strain surface GP. The standard, licensed rVSV∆G-ZEBOV-GP vaccine dose (1 mL, ≥72 million pfu by intramuscular (IM) injection) will be used in this study. The use of the VSV vector offers multiple advantages, including low seroprevalence of pre-existing vector-specific immunity (supported by the removal of the GP), no host integration of antigen RNA, and ability to accommodate large transgenes. Vaccination with rVSVΔG-ZEBOV-GP provides protection against Ebola virus disease (EVD) caused by the Zaire strain. Primary data analysis from the phase III ring vaccination study, which recruited contacts and contacts-of-contacts of Ebola virus, demonstrated 100% vaccine efficacy against EVD in 2041 subjects who were vaccinated immediately on contact in the 2015 West Africa outbreak and this vaccine has subsequently been used to mitigate further outbreaks in Central Africa.^3^ The vaccine does not confer protection from other species of Ebola virus and the relative contributions towards Zaire strain-specific immunity from innate, humoral and cell-mediated responses are unknown, and defined immune correlates of protection from vaccination or natural infection remain elusive. Data from rVSVΔG-ZEBOV-GP clinical trials has often characterised humoral immune responses using the quantitation of serum IgG-antibody to purified Kikwit ZEBOV GP by validated ELISA (GP-ELISA), which bore close statistical correlation with functional performance of antibody-mediated neutralisation measured by plaque reduction neutralisation titre (PRNT).^4^,^6^ Sero-response criteria have been defined as >2-fold increase in GP-ELISA antibody from baseline and ≥200 EU/mL at any time post vaccination (met by 94% of vaccine trial participants) and ≥4-fold increase from baseline at any time post vaccination by PRNT (met by 80% of vaccine trial participants). A more detailed breakdown of the immunogenicity responses in clinical trials can be seen in the summary of product characteristics document for rVSVΔG-ZEBOV-GP.^7^

### SARS-CoV-2 virus and mRNA vaccines for COVID-19

SARS-CoV-2 is a respiratory virus that emerged in China in November 2019 and a global pandemic was declared on 11 March 2020.^8^ Since February 2022, the Omicron variant has accounted for 98% of clinical isolates and is likely to be the basis for the emergence of further SARS-CoV-2 variants.^9^ To date, there have been 766 million confirmed cases of SARS-CoV-2, 6.9 million confirmed deaths and 13.4 million vaccine doses administered worldwide.^10^ Moderna and Pfizer–BioNTech manufactured messenger RNA (mRNA) COVID-19 vaccines were the commonly used products in the global effort to vaccinate against SARS-CoV-2.^11^ Comirnaty (Pfizer–BioNTech) encodes for full length spike protein with two-point mutations to proline in the central helix to lock spike into the prefusion conformation. Clinical trials of Comirnaty reported a good safety profile and vaccine efficacy of 95% in one clinical trial where 21 720 participants received the vaccine.^12^

### Rationale for the proposed study

Vaccines for the prevention of severe disease caused by Ebola virus and SARS-CoV-2 virus are routinely offered to adults at higher risk of exposure in the African settings. For protection from Ebola virus, the main target populations are epidemiologically identified and include healthcare workers, refugees and people living in outbreak zones. These target populations are also routinely offered vaccines for other vaccine-preventable diseases. However, there are currently no data on whether the co-administration of rVSVΔG-ZEBOV-GP with other vaccines has an acceptable profile of reactogenicity and antigen-specific immunogenicity, and concomitant use of rVSVΔG-ZEBOV-GP with other vaccines is not recommended. Clinical trial data on the co-administration of rVSVΔG-ZEBOV-GP with other vaccines prioritised for the same population, and especially in the context of a new era in mRNA vaccine technology, would have significant relevance to how protection from other diseases can be provided at a single clinic visit alongside the rVSVΔG-ZEBOV-GP vaccine. This, in turn, has the potential to improve vaccine coverage, the efficiency of vaccine policy and logistics, as well as being the first step in the evaluation of VSV-vectored vaccine technology with lipid-enveloped mRNA platforms that are going to be increasingly used in other formulations against other/future infectious disease targets.

### Significance of selected topic

There are no preliminary data in this first-of-kind study. The advent of the SARS-CoV-2 virus and COVID-19 pandemic signalled the accelerated development of lipid-nanoparticle mRNA vaccine technology with great success. The mRNA platform is highly adaptable to new disease targets, including cancer and infectious disease pathogens, and a multitude of vaccine candidates are currently in development for outstanding global health priorities that have eluded pre-pandemic technologies. However, very little is known about using mRNA technologies in combination with, or concurrent to, other vaccine technologies and whether there are any adverse signals associated with safety or antigen-specific immune response/protection. We are only now beginning to answer this important data gap. Earlier trials have focused on using SARS-CoV-2 mRNA vaccines in combination with adjuvant protein vaccines for seasonal influenza (and herpes zoster virus, in setup) and have reported favourable safety and humoral immune responses. This has resulted in the recommendation to include the concurrent vaccine dose administration within the annual seasonal vaccination schedule of UK nationals.^13^ These study data have significantly supported public confidence in accepting two licensed vaccines at the same time, and significant efficiency gains in vaccine coverage and public protection. The concurrent administration of mRNA vaccines with attenuated or replication-incompetent viral-vectored vaccines has not, to our knowledge, been assessed although many trials reported favourable safety and immunogenicity with heterologous combinations of these vaccine platforms for protection against COVID-19.^14^,^16^ Since existing mRNA COVID-19 vaccines and new mRNA constructs against other infectious diseases are expected to become routine immunisations for key members of the population, such as healthcare workers and older adults, research is needed to inform whether concurrent or co-administration with other vaccines is possible. For African populations, the risk of disease outbreaks from Ebola remains high and the future use of rVSVΔG-ZEBOV-GP is expected, along with other measures, to mitigate the risks to healthcare workers and the wider public. Assessing the safety and immunogenicity of rVSVΔG-ZEBOV-GP vaccine in the context of expected gains in public confidence and policy implementation with co-administration with mRNA vaccine technologies is a major subject of interest for African populations and preparedness for future disease outbreaks.

## Methods and analysis

We used the Standard Protocol Items: Recommendations for Interventional Trials checklist when preparing the present protocol report.^17^

### Trial design and setting

This is a single-centre, randomised, participant-blinded, vaccine safety and immunogenicity study in healthy adults living in Rwanda. The EbolaCov trial aims to inform whether the Ebola vaccine rVSVΔG-ZEBOV-GP can be administered concurrent to a Pfizer–BioNTech COVID-19 booster dose without an unacceptable increase in reactogenicity and/or loss of humoral immunogenicity to Ebola vaccine antigen.

This trial is being conducted at the research and healthcare facility the Center for Family Health Research in Kigali, Rwanda, in collaboration with Rwanda Biomedical Centre, the national central health implementation agency for Rwanda.

### Objectives and outcomes

The primary safety objective is to determine the frequency and severity of severe solicited systemic adverse reactions within 1 week of concurrent vaccine dose administration, compared with giving the rVSVΔG-ZEBOV-GP vaccine alone. Participant-reported reactogenicity data will be entered via an e-diary or a backup paper diary. Data includes solicited adverse events (AEs), including injection site reactions (pain, tenderness, erythema, swelling, axillary swelling or tenderness), as well as systemic symptoms (headache, malaise, myalgia, nausea, vomiting and fever). AEs of special interest include arthritis, synovitis, joint effusions, temporomandibular joint syndrome, tendonitis, mucosal or skin lesions and postvaccination dermatitis. All AEs will be graded in severity by the participant and then reviewed by the study team. Relationship to the trial vaccines will be determined by a study physician.

The primary immunogenicity objective is to measure serum anti-GP antibody responses 28 days after concurrent vaccine dose administration, compared with giving the rVSVΔG-ZEBOV-GP vaccine alone. This outcome will be measured using day 28 serum anti-GP antibody responses assessed by commercially available ELISA.

Secondary objectives include longitudinal assessment of humoral response to rVSVΔG-ZEBOV-GP and SARS-CoV-2 when co-administered with Pfizer–BioNTech COVID-19 booster vaccination, compared with giving the rVSVΔG-ZEBOV-GP vaccine alone, and assessment of SARS-CoV-2 anti-spike protein specific geometric mean titres (GMT) when co-administered with rVSVΔG-ZEBOV-GP vaccine. Secondary immunogenicity outcome measures include day 28 and day 180 anti-GP and anti-spike GMT measured by commercially available ELISA.

We also aim to evaluate the perceptions and acceptability of concurrent vaccination among healthcare workers and volunteers who took part in the study using transcripts from semi-structured interviews (or focus groups) conducted for this purpose.

### Recruitment and eligibility

We aim to primarily recruit participants who are current healthcare workers, although the study will be open to all eligible members of the public. Study participants will be self-selected individuals responding to invitations to consider taking part. Recruitment/engagement will include staff email contact, conference/meeting presentations and other methods using Rwanda National Ethics Committee (RNEC) approved methods and materials. A total of 72 participants is required. Participants must be able to consent, healthy, aged 18–50 years, and should have already received a WHO approved primary COVID-19 immunisation course for inclusion into the study.

Participants will receive information via an RNEC-approved study information booklet (SIB) followed by a discussion with trained study staff, who will then gain informed consent. The SIB is available in both English and Kinyarwanda, the national language of Rwanda, and the informed consent discussion will be in the language of choice of the participant. Witnessed consent with a thumbprint as confirmation of consent will be available for illiterate individuals. RNEC-approved copies of the SIB and informed consent form are available in the online supplemental material (English and Kinyarwanda versions). Eligibility will be assessed during the screening visit based on medical and vaccination history, physical examination, vital signs and pregnancy and HIV point-of-care testing. Demographic data will also be collected at this point.

**Figure 1:**
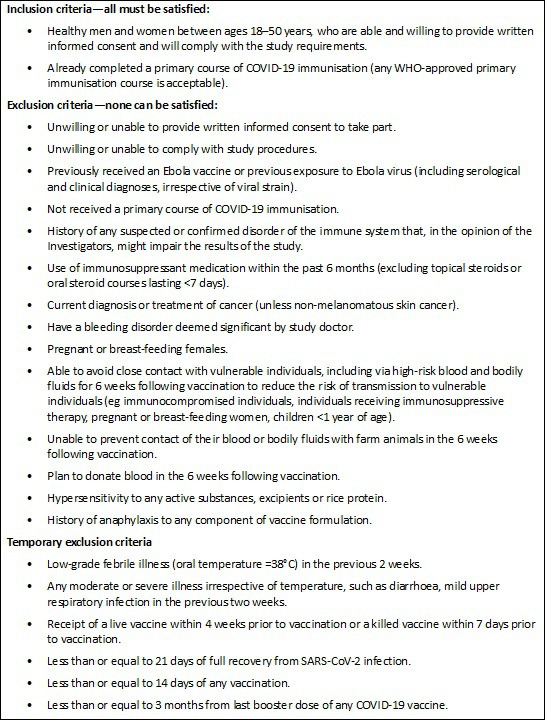
EbolaCov eligibility criteria.

Eligibility criteria broadly reflect the summary of medicinal product characteristics for the licensed rVSVΔG-ZEBOV-GP vaccine and the emergency use authorisation requirements for the Pfizer–BioNTech vaccine when used to control the recent COVID-19 pandemic. These are outlined in figure 1. Exclusion criteria include any condition that may impair the immune response to vaccination or would mean receipt of either vaccine would be high risk for the participant (figure 1). Enrolment may be delayed if temporary exclusion criteria likely to bias the results are met (figure 1).

### Study interventions

This study is not yet open to recruitment. The planned study start date is 1 September 2025, with estimated completion in May 2026.

Participants who fulfil eligibility criteria will be randomised using the data management software REDCap in a 1:1 ratio to the following group allocations:

- Group 1 (n=36) will receive a single dose of rVSV∆G-ZEBOV-GP vaccine by IM injection (1 mL) to the deltoid muscle of the non-dominant arm concurrent with an IM injection of placebo (1 mL saline solution) to the deltoid muscle of the dominant arm on day 0, followed by the offer of a single-dose of Pfizer–BioNTech COVID-19 vaccine by IM injection to the deltoid muscle of the non-dominant arm 6 months later.
- Group 2 (n=36) will receive a single dose of rVSV∆G-ZEBOV-GP vaccine by IM injection (1 mL) to the deltoid muscle of the non-dominant arm concurrent with a single-dose of Pfizer–BioNTech COVID-19 vaccine by IM injection to the deltoid muscle of the dominant arm on day 0.

All participants will undergo follow-up over 6 months; a screening visit, a vaccination visit within 5 days of screening (day 0), a reactogenicity diary review visit (day 7) and two follow-up visits with blood donations at day 28 and day 180 post vaccination. There may be additional safety visits if required. A participant is considered enrolled at the time of receiving the first dose of the vaccine and there is no replacement of participants who withdraw from the study early.

An outline of study procedures is shown in table 1.

**Table 1:** Study schedule

Activity	Screening	Day 0*	Day 7† (+ 4 days)	Day 28 (±4 days)	Day 180 (±14 days)
Informed consent	X				
Eligibility assessment	X				
Medical history check / assessment	X	X	X	X	X
HIV point-of-care test	X				
Urine pregnancy test (if appropriate)	X				
Vital signs	X	X		X	X
Serology sampling (20 mL blood)		X		X	X
Randomisation		X			
Review of any new concomitant medications		X	X	X	X
rVSV-ZEBOV-GP and COVID-19 booster or placebo administration (as per study group)		X			
Semi-structured interviews					X
Unblinding					X
COVID-19 vaccination offer (as per study group)					X

* Must be within 5 days of screening

† Day seven visit can be completed by telephone for participants using the e-diary

### Blood sampling

A 20 mL blood sample will be collected at enrolment, day 28 and day 180 for laboratory analyses to assess the objectives defined above and antibody responses to vaccination only, and there is no storage of cells or DNA. Laboratory analyses will be performed at the research site (Center for Family Health Research, Kigali) or at the Rwanda Biomedical Centre reference laboratory in Kigali. The GMT and antigen-specific antibody titre will be measured by commercial GP-ELISA 28 days and 180 days after vaccination.

### Semi-structured interviews (focus groups)

A subset of 5–20 participants and 5–20 healthcare professionals will be invited to take part in semi-structured interviews in groups of 10 (a focus group) to receive feedback on the perceptions of concurrent vaccination and taking part in the study to explore perceptions, and acceptability of concurrent vaccination with two vaccines versus individual vaccination.

### Governance

An independent data safety monitoring committee (DSMC) will support this study by providing independent oversight of safety and trial conduct in accordance with a predefined study-specific DSMC charter. The committee will have access to unblinded immunogenicity and safety data, including at 7 days and 28 days following vaccination and following any significant AE. The DSMC will make recommendations on whether there are any ethical or safety reasons why the trial should not continue.

The quality assurance manager will conduct internal audits to check that the trial is being conducted, data recorded, analysed and accurately reported according to the protocol and in compliance with International Conference on Harmonisation guidelines for good clinical practice (ICH GCP), meeting the requirements of the RNEC and Rwandan Food and Drug Authority (FDA). The audits will also include laboratory activities according to an agreed audit schedule, taking into consideration the guidelines for GCP in the laboratory. The internal audits will supplement the external monitoring process and will review the processes not covered by the external monitor.

### Safety

Both vaccines for use in this study have been demonstrated to have good safety profiles in previous studies when administered separately; however, there are no data on safety when these two vaccine platforms are used concurrently. General risks associated with the trial may include side effects associated with phlebotomy and those outlined in the summary of product characteristics of each vaccine. All solicited and unsolicited AEs after vaccination will be monitored throughout the study. The chief investigator and the sponsor office will be notified within 24 hours of the study team discovering the onset of the occurrence of a serious AE (SAE). RNEC and the Rwandan FDA will be notified of an SAE within 7 days of the study team being made aware of the event.

Any pregnancy occurring during the clinical study and the outcome of the pregnancy will be recorded and followed up for congenital abnormality or birth defect. Pregnancy notification and follow-up reports will be provided to the DSMC.

### Sample size

No studies have analysed the concurrent administration of rVSV-vector and lipid-enveloped mRNA vaccine technologies to provide a basis for power calculations.

Published data from clinical trials of rVSVΔG-ZEBOV-GP showed an anti-GP serum antibody GMT of 994.7 (915–1081, SD 38.9)^5 7^ 28 days after vaccination. These data help to design a study to investigate humoral immune responses to vaccination in the context of concurrent dosing with an mRNA COVID-19 product. A standard significance (alpha) value of 5% and 80% power were applied for a statistically significant likelihood of detecting any inferior GMT responses to rVSV-ZEBOV-GP between study groups. A 25% non-inferiority margin was selected, in line with the previous co-administration studies.^12^ Pragmatically, it enables a deliverable, while clinically meaningful, phase IV co-administration study that is the first examination of rVSV-ZEBOV-GP and mRNA vaccine technology as co-administration where Ebola immune correlates of protection are unknown. A 10% non-inferiority margin would require large cohort sizes that exceed operational capacity (with this margin the requirement would be n=188 per group). A cohort size of 32 was calculated in line with these parameters.^18^ An additional 10% recruitment margin is added as contingency for participant withdrawal or dropout before day 28. This finalises each cohort size at 36 participants, totalling 72 participants.

The study population size is not powered for comparative serum anti-spike protein antibody responses due to impractical cohort sizes and wider variation in GMT distribution and CIs: a statistically significant non-inferior response 28 days after third dose boosting (Pfizer–BioNTech; GMT 1789, 1520–2107, SD 134.5) would require a cohort size of n=359.^16^ The sample sizes reflect operational delivery expectations and also afford stability of estimates in reactogenicity and immunogenicity data not formally powered for.

### Statistical analysis plan

Analyses of both the intention-to-treat and per-protocol populations will be presented. Statistical analyses will be descriptive in nature (counts/proportions, or GMT where appropriate). Any comparative statistics (χ^2^/Fisher’s-exact or paired/unpaired t-test where appropriate) and analyses of exploratory/longitudinal data will be post-hoc analyses.

The full details of the analysis will be provided in the statistical analysis plan which will be finalised prior to unblinding and database lock.

Interview transcripts will be analysed qualitatively.

### Data management plan

All data are pseudoanonymised at source. At screening, all participant data and samples will be identified by a study-specific participant number and/or code. Data will be collected via an electronic case report form using REDCap. These data will be stored on secure, encrypted servers at Rwanda Biomedical Centre with password-controlled access to authorised study team members only.

### Patient and public involvement

There are no patients involved in this trial. Healthy adults will be recruited as participants. There was no public involvement in the development of the research questions or study design, but attitudes and perspectives on this approach will be captured by the qualitative focus group activity as a part of the trial.

## Ethics and dissemination

This study protocol has been reviewed and approved by the RNEC (reference RNEC442/2024) and the University of Birmingham (reference ERN_2661-Jun2024). The study will be conducted in accordance with the principles of the Declaration of Helsinki and relevant regulations of the ICH GCP. The sponsor is the University of Birmingham who will provide indemnity for compensation for harm arising as a consequence of participation of the research subjects in the trial. This trial is registered on the Pan African Clinical Trials Registry (PACTR202407764378004) and ClinicalTrials.gov (NCT06587503).

Informed consent will be obtained from all participants. Participation in the trial is strictly voluntary, and withdrawal can occur at any point in the trial. Participants will be reimbursed a small fee to cover expenses for attending trial visits.

Results will be published in a peer-reviewed medical journals and presented at national and international conferences. All publications (eg, manuscripts, abstracts, oral/slide presentations and book chapters) from one study will be reviewed by each investigator prior to submission.

## Supplementary material

10.1136/bmjopen-2025-102898 online supplemental file 1

10.1136/bmjopen-2025-102898 online supplemental file 2

## References

[1] Kaner J, Schaack S (2016). Understanding Ebola: the 2014 epidemic. Global Health.

[2] World Health Organization (2025). Mpox: Multi-country external situation report no. 47.

[3] Henao-Restrepo AM, Camacho A, Longini IM (2017). Efficacy and effectiveness of an rVSV-vectored vaccine in preventing Ebola virus disease: final results from the Guinea ring vaccination, open-label, cluster-randomised trial (Ebola Ça Suffit!). Lancet.

[4] Boum Y, Juan-Giner A, Hitchings M (2020). Humoral and cellular immune response induced by rVSVΔG-ZEBOV-GP vaccine among frontline workers during the 2013–2016 West Africa Ebola outbreak in Guinea. Vaccine (Auckl).

[5] Kennedy SB, Bolay F, Kieh M (2017). Phase 2 Placebo-Controlled Trial of Two Vaccines to Prevent Ebola in Liberia. N Engl J Med.

[6] Samai M, Seward JF, Goldstein ST (2018). The Sierra Leone Trial to Introduce a Vaccine Against Ebola: An Evaluation of rVSV∆G-ZEBOV-GP Vaccine Tolerability and Safety During the West Africa Ebola Outbreak. J Infect Dis.

[7] Summary of Product Characteristics - Ervebo European Medicines Agency.

[8] World Health Organization (2020). WHO Director-General’s opening remarks at the media briefing on COVID-19.

[9] World Health Organization (2023). Statement on the update of WHO’s working definitions and tracking system for SARS-CoV-2 variants of concern and variants of interest.

[10] World Health Organization (2023). Coronavirus disease (COVID-19) pandemic; Numbers at a glance.

[11] Data OWi (2024). COVID-19, vaccinations (broken down by manufacturer.

[12] Polack FP, Thomas SJ, Kitchin N (2020). Safety and Efficacy of the BNT162b2 mRNA Covid-19 Vaccine. N Engl J Med.

[13] Lazarus R, Baos S, Cappel-Porter H (2021). Safety and immunogenicity of concomitant administration of COVID-19 vaccines (ChAdOx1 or BNT162b2) with seasonal influenza vaccines in adults in the UK (ComFluCOV): a multicentre, randomised, controlled, phase 4 trial. Lancet.

[14] Liu X, Shaw RH, Stuart ASV (2021). Safety and immunogenicity of heterologous versus homologous prime-boost schedules with an adenoviral vectored and mRNA COVID-19 vaccine (Com-COV): a single-blind, randomised, non-inferiority trial. Lancet.

[15] Stuart ASV, Shaw RH, Liu X (2022). Immunogenicity, safety, and reactogenicity of heterologous COVID-19 primary vaccination incorporating mRNA, viral-vector, and protein-adjuvant vaccines in the UK (Com-COV2): a single-blind, randomised, phase 2, non-inferiority trial. Lancet.

[16] Munro APS, Janani L, Cornelius V (2021). Safety and immunogenicity of seven COVID-19 vaccines as a third dose (booster) following two doses of ChAdOx1 nCov-19 or BNT162b2 in the UK (COV-BOOST): a blinded, multicentre, randomised, controlled, phase 2 trial. Lancet.

[17] Chan A-W, Tetzlaff JM, Gøtzsche PC (2013). SPIRIT 2013 explanation and elaboration: guidance for protocols of clinical trials. BMJ.

[18] Julious SA (2004). Sample sizes for clinical trials with normal data. Stat Med.

