# Supplementary material for: Safety and immunogenicity of rVSVΔG-ZEBOV-GP vaccination when dosed concurrent with mRNA COVID-19 vaccine booster doses in healthy African adults (EbolaCov): protocol for a phase IV, single-centre, single-blinded, randomised controlled trial
Source: BMJ Open. 2025 Sep 21;15(9):e102898. doi: 10.1136/bmjopen-2025-102898 (PMC12458633; doi:10.1136/bmjopen-2025-102898)
Supplement: online supplemental file 1 [file bmjopen-15-9-s001.pdf]

EbolaCov

EbolaCov: A phase IV, single-centre, single-blinded, randomized controlled trial to assess safety and immunogenicity of rVSVΔG-ZEBOV-GP vaccination when dosed concurrent with mRNA COVID-19 vaccine booster doses in healthy African adults.

STUDY INFORMATION BOOKLET AND INFORMED CONSENT FORM

You are invited to take part in a clinical trial to assess the safety and immune response to Ebola and COVID-19 vaccines when given at the same time. The study is being run by the University of Birmingham, Rwanda Biomedical Center and the Center for Family Health Research.

Before you decide whether to take part, it is important for you to understand what the study is about and what participation would involve. Please take time to read the information carefully and discuss with others if you wish. If anything is unclear or you would like further information, please contact the study team (details below). Thank you for taking the time to consider taking part in the study.

Contact details:

Center For Family Health Research (CFHR)-Project San Francisco (PSF).
Rwanda Zambia Health Research Group (RZHRG)
KK19Av 57, PO Box 780, Kigali-Rwanda
www.rzhrg.org

Rwanda National Ethics Committee

Approval Date: ________________

Expiration Date: ________________

What is the purpose of this research trial?

We want to look at the safety and immune response to Ebola vaccination when this vaccine is given at the same time as a COVID-19 vaccine. The Ebola vaccine, rVSVΔG-ZEBOV-GP, has been shown to be safe and effective for protection against Ebola virus disease (EVD), and was approved for use in 2019. More recently, we have developed new vaccines for protection against severe COVID-19 disease. This trial has been designed to see how well the body responds to the Ebola vaccine when it is given at the same time as an mRNA COVID-19 vaccine. We will look at both safety and the scale of immune response (antibody levels) to see if this approach means we can have more efficient vaccination programmes in the future.

Summary of the trial

We aim to recruit 72 healthy volunteers aged between 18 and 50yrs.

Study volunteers will be randomly assigned to either (a) receive a single dose Ebola vaccine in one arm, and a single dose of a COVID-19 vaccine in the other arm, or (b) receive a single dose Ebola vaccine in one arm, and a placebo in the other arm.

You will not get to choose which study group you are assigned to, and participation in this study will be for 6-months in total. At the end of the study, you will get to find out which vaccines you received at the start of the study and be offered a COVID-19 vaccine if you did not have one earlier.

Every participant will we asked to complete a record of any side effects of 7-days after vaccination and attend the clinical trial site on 4 to 5 occasions over 6-months so that the study team can review your welfare and collect a small sample of blood (20mLs) to measure antibody levels at important time points after vaccination.

Which vaccines are used in this trial?

We will be using two licensed vaccines:

- The Merck Ervebo® Ebola Vaccine (rVSV-ZEBOV-GP), sold under the brand name Ervebo. This is a live vaccine that uses a genetically carrier virus (called vesicular stomatitis virus, or VSV) to deliver the genetic code for the body to produce Ebola protein and trigger an immune response that protects from the real Ebola virus in the future. It is not possible to become infected with Ebola from the vaccine, and this vaccine has undergone extensive testing in earlier clinical trials and has been shown to be safe and effective and was licenced in 2019.

- Pfizer–BioNTech COVID-19 vaccine, sold under the brand name Comirnaty. This is an mRNA-based SARS-CoV-2 vaccine currently in use as a booster dose in Rwanda. This vaccine type uses a fat bubble to deliver the genetic code for the body to produce SARS-CoV-2 protein and trigger an immune response that protects from severe disease in the future. It is not possible to become infected with coronavirus from the vaccine, and this vaccine has also undergone extensive clinical trial testing and has been shown to be safe and effective and was first licenced in late 2020.

A great many members of the public have already had both these vaccines, at different times in their lives, and there have been no safety concerns raised. The focus of this study is to examine for any effects on safety and/or the immune (antibody) response to the Ebola vaccine, when an mRNA COVID-19 vaccine is **given at the *same* time.**

Do I have to take part?

No, this is voluntary. It is up to you to decide whether to take part. If you decide to take part, you will be given this information sheet to keep (or have an electronic copy) and will be asked to sign the consent form at the end of this document. If you decide to take part, you can change your mind at any time and do not need to give any reason.

Who is suitable to take part in this trial?

We are looking for healthy volunteers aged 18-50 years. In addition,

- You must be willing to share your medical history
- You must be able to attend all 3 visits in person over the 6-months
- You must not have previously had an Ebola vaccine or previous exposure to the Ebola virus
- You must have already received at least two doses of a COVID-19 vaccine, and your most recent dose must be more than 3 months ago
- You must not be pregnant, breastfeeding or planning to be pregnant in the next 6-months
- You must not have any significant problems with your immune system, or taken any immunosuppressant medication in the last 6-months
- You must have no current diagnosis of cancer (unless non-melanoma skin cancer)
- You must have no significant bleeding disorders
- You must avoid close contact with vulnerable individuals such as those with impaired immune systems, young children (aged <1yr), and pregnant and/or breastfeeding women for 6-weeks following vaccination
- You must avoid contact with farm animals for 6-weeks following vaccination
- You must not have allergies to any of the vaccines or rice protein
- You must agree to not donate blood in the 6-weeks following vaccination
- You must not have had any other vaccines in the 14-days prior to entering the study
- You must not have had symptoms of a COVID infection in the last 21-days

What happens if I want to take part?

Please contact a member of the study team if you are considering taking part. You will then be invited to meet with a member of the study team to discuss the study in more detail and answer any questions that you have. Once you have had all the information you need and time to consider taking part in the study, you will be asked if you wish to proceed to the next steps of signing the consent form and allowing a member of the study team to assess whether it is safe and suitable for you to take part. We call this the **screening visit (called V0)** and this is where we start to collect information from you. The purpose of screening assessments is to ensure that you will not be taking on an extra risk to your health.

What information do you collect about me if I take part in trial assessment (screening)?

- We ask questions about you, such as your date of birth, race, contact details (home address, phone and email contact), ask questions about your medical history where this is relevant to taking part in the study, and your availability to attend clinic appointments.
- We measure your vital signs (heart rate, blood pressure, body height, body mass)
- We undertake a simple physical examination
- We take a fingerpick of blood and undertake a point-of-care test for HIV
- We ask for a urine sample to undertake a pregnancy test for any females of childbearing potential

Once the study team has been able to determine if you can take part, then you will be invited to the first visit (called V1).

What can I expect if I take part in the trial?

Each volunteer will be invited to attend the clinic on 4 occasions after screening, as well as collect data at home and to keep in contact with the study team throughout the study.

The first visit (or V1) is within 5-days of screening

- We check you are still willing and able to take part in the study
- We collect 20mLs of blood to measure pre-vaccination antibody levels
- We then use a computer to randomly select which study group you enter – neither you or the study team can choose which study group you are allocated to
- You then receive two injections, one given shortly after each other; both by needle and syringe and one injection will be into the upper muscle area on one arm, the other injection will be into the upper muscle area on the opposite arm
- You need to wait and be observed by the study team for at least 15 minutes after vaccination
- You are considered enrolled into the study when you receive the first injection
- You will know that one injection is a single dose of the Ebola vaccine - but you will not know which arm this was given to
- You will know that one injection is either a single dose of the mRNA COVID-19 vaccine or a placebo (medical grade saline solution), and you will not know which arm it was given to
- You receive training on how to collect data on the electronic diary and paper records, as well as contact details for the study team, and be given a thermometer and measuring tape to take home

After the first visit you will complete an electronic or paper diary record for the next 7-days that describes, every day, any side effects to vaccination. These include;

- Local reactions at the site of both injections; presence, severity and duration of any injection site pain, redness, swelling/induration or armpit swelling/tenderness
- Systemic reactions; recording of oral temperature and the presence, severity and duration of any headache, fatigue, myalgia, arthralgia, nausea/vomiting, chills or abdominal pain
- Any additional concerns for pain and/or swelling around any joints (including around the jaw and tendons), rashes or other skin lesions (including inside the mouth)
- Any other symptoms or concerns

The second visit (or V2) will be 7-11 days after vaccination

- We review any side effects to vaccination which you recorded on the electronic or paper diary that we give you to complete; this can be done by telephone if the electronic diary is complete or in person if you prefer to use the paper diary

The third visit (or V3) will be 28-days (+/- 4-days) after vaccination

- We check you are still willing and able to take part in the study; this includes any new information about your health
- We measure your vital signs (heart rate, blood pressure)
- We collect 20mLs of blood to measure post-vaccination antibody levels

The fourth and final visit (or V4) will be 6-months (+/- 14-days) after vaccination

- We check you are still willing and able to take part in the study; this includes any new information about your health
- We measure your vital signs (heart rate, blood pressure)
- We collect 20mLs of blood to measure post-vaccination antibody levels
- We tell you which vaccines you had at the start of the study
- You then have the option to have a single dose of COVID-19 vaccine if you did not receive one at the start of the study

What are the risks of taking part in this trial?

The study team are primarily here for your safety. The risks or taking part can be summarised as;

- **Blood sampling** uses a needle and syringe and can commonly cause transient pain/discomfort and sometimes some bruising. We take only 20mLs of blood on each visit (this is about the same as 5 teaspoons in volume) and the total collection of 60mLs of blood over the 6-months of the study poses no harm to your health.

- **Vaccinations** commonly cause transient pain/discomfort and sometimes some bruising at the site of injection. Other local reactions can include mild and transient redness or swelling, and this should fully resolve within a few days. Occasionally some individuals feel systemically unwell with fever or aches/pains, but this is mild and transient. Both the Ebola and COVID-19 vaccines used in this study are licenced products, which means they have completed extensive evaluation through clinical trials and have been approved for use by independent regulatory authorities.

- **Serious reactions to vaccination** are rare events and when they do occur this is most commonly in the context of an allergic reaction. Life-threatening anaphylaxis reactions occur in approximately 1 per million vaccine doses, and usually soon after vaccination when the study team is still with you and are trained to help if needed. mRNA COVID vaccines are rarely associated with facial weakness and/or inflammation of the heart (approximately 1 in 10,000 risk).

What are the benefits of taking part in this trial?

Both vaccines have been shown to be effective in preventing disease. However, we do not know if receiving both vaccines at same time changes the quantity of antibody made to one or both vaccines. This is one of the reasons we are doing this study.

Additional benefits can include the experience of taking part and contributing to important science for future individuals who might benefit from this work, and you will be learning about your own health.

Do I have to pay for taking part in this trial?

You do not have to pay for taking part in this study. **You will be reimbursed 12,000 rwf** for every completed visit for your time and any expenses incurred in taking part in the study.

What tests are done on my blood samples?

Your samples are tested at the study site in Kigali and, if you agree, at laboratories of project partners in Rwanda and the United Kingdom. We conduct safety assessments and measure the quantity of antibody in response to vaccines used in this study only. We do NOT undertake any genetic tests, store or use your cells, or undertake any analysis other than the assessment of safety and antibody response to vaccination.

Do I get access to extra medical treatment from being in the trial?

No. You do not lose any legal or medical rights by taking part in the study, but there will be no extra access to healthcare because of taking part in the trial. If you have private medical insurance, you may wish to check with your insurer before taking part in this study. If you become unwell during the trial, the study team will be available to investigate if this is in relation to you taking part in this study. Should you need medical care then the study team can help to arrange this.

What if new information becomes available?

If new information from other studies that use the same vaccines comes to the attention of the study team, we will tell you about it and discuss these details and what it might mean for your participation.

Are there interviews about taking part?

For some study volunteers we ask your thoughts and opinions about this research. This is optional and a member of the study team will discuss this separately with you if invited to help this way at the end of the study. Interviews are confidential and you can still take part in the study without taking part in this.

Do I have to complete the trial?

No. You are free to change your mind and withdraw from the trial at any time. We would keep the information and samples collected from you up until when you tell us that you wish to withdraw from the study, but not ask anything more from you unless there was a safety concern. Your participation in this trial may also be stopped at any time by the study team for safety reasons.

The only time we need to keep in contact with you after leaving the trial was if there was a safety reason of if you were to become pregnant and to ensure the pregnancy went normally.

Study participant initials/thumbprint: _______________

What if something goes wrong?

The study team recognises the important contribution that volunteers make to medical research and make every effort to ensure your safety and well-being. The University of Birmingham, as the research Sponsor, has arrangements in place in the unlikely event that you suffer any harm as a direct consequence of your participation in this trial. In the event of harm being suffered, while the Sponsor will cooperate with any claim, you may wish to seek independent legal advice to ensure that you are properly represented in pursuing any complaint. The study team can advise you of further action and refer you to a doctor if necessary.

Would my taking part in this trial be kept confidential?

Yes. Your participation, your data and your samples are all kept confidential. All information that is collected about you is coded with a unique study number that is used in all your records. The register of how each code is connected to your true identity is stored securely, separately and is not shared outside if the team authorised to work on this study. All information and samples taken from you will be anonymised as soon as possible using your assigned clinical trial number.

Data are stored on secure, encrypted severs, and access will be restricted to members of the study team with password-restricted controls. The electronic diary is sent to you by email to complete online and your email address will be stored on a secure server.

To ensure that the study is being conducted correctly, representatives of the study Sponsor (University of Birmingham) and regulatory authorities (Rwanda Food and Drug Administration) and Rwanda National Ethics Committee can request access to study records without violating your confidentiality.

What happens to the results of this study?

The results of this study will be published in scientific journals, but you cannot be identified in any report or publication. Once the study is published, we will send out a summary of the results to all study participants. You will not have access to your personal results.

Who is funding the study?

The study is funded by the Merck Sharp & Dohme (MSD) and the Merck Investigator Studies Program (MISP) programme.

Study participant initials/thumbprint: _______________

Who can I contact if I have any questions, comments, complaints, or concerns?

We sincerely hope that you will have a good experience in working with the study team. If you wish to contact any of us for any reason, please contact any of the following study team members;

- Principal Investigator **Dr Julien Mutagisha Nyombayire,** Center for Family Health Research, on email jnyombayire@rzhrg-mail.org
- Co-Investigator **Prof Claude Mambo Muvunyi**, Rwanda Biomedical Center, on email claude.muvunyi@rbc.gov.rw or phone +250788493814.
- Chief Investigator **Dr Christopher Green**, University of Birmingham, UK, on email c.a.green.2@bham.ac.uk

If you have any questions concerning your rights as a participant in this study, please contact the following representatives of the Rwanda National Ethics Committee;

- Chairperson of Rwanda National Ethics Committee, **Dr. Vedaste Ndahindwa** on email ************ or phone ***********.
- Secretary of Rwanda National Ethics Committee , **Dr. Marie Françoise Mukanyangezi** on email **************** or phone ************

Study participant initials/thumbprint: _______________

UNIVERSITY OF BIRMINGHAM

CENTER FOR FAMILY HEALTH RESEARCH

rbc Rwanda Biomedical Centre
Healthy People, Wealthy Nation

EbolaCov

EbolaCov: A phase IV, single-centre, single-blinded, randomized controlled trial to assess safety and immunogenicity of rVSVΔG-ZEBOV-GP vaccination when dosed concurrent with mRNA COVID-19 vaccine booster doses in healthy African adults.

SIGNATURE PAGE OF THE INFORMED CONSENT FORM

Statement of participant:	Initial/thumb print each box
I confirm I have read and understood this study information booklet and informed consent form. I have had the opportunity to consider the information, ask questions, and have had these questions answered to my satisfaction.	
I understand my participation is voluntary and I am free to withdraw any time without giving reason, without my medical care or legal rights being affected	
I understand the data collection during the study, including my medical information, may be looked at by individuals from the sponsor, its representative and/or the regulatory authorities, where it is relevant to me taking part in this research. I give permission for these individuals to have access to my records.	
I understand that my personal, coded data will continue to be processed after the completion of the study or after I withdraw from the study if necessary, for reasons of public interest in public health, for archiving purposes in the public interest, for scientific research purposes, or for statistical purposes.	
I understand the information collected about me will be used to support other research in the future and may be shared anonymously with other researchers.	
I agree to the use of my samples for the purposes described in this information booklet and informed consent form.	
I understand that I will receive a copy of this signed study information booklet and informed consent form. If I decline to take a copy of the informed consent form, the investigator may keep it for me confidentially at the study site.	

I agree to take part in the study of my own free will	
[**Optional**] I agree that any leftover blood samples from this study can be used for further vaccine-related research purposes and other immunity studies. This may include sending samples to laboratories at partner organisations in Rwanda (RBC) or the United Kingdom (University of Birmingham). I understand that my identity would remain confidential, that if I decline this optional consent that I can still take part in the trial, and that future research would be subject to regulatory approvals.	

**Name of study participant** (printed)	**Signature of study participant**	**Date and time** (DD/MMM/YY) and (24hrs)

<u>Statement of Impartial Witness (if the study participant is illiterate)</u>

I sign here as a witness to the consent process. I have participated in the discussion and witnessed the participant voluntary consent to study participation.

**Name of independent witness if required** (printed)	**Signature of witness**	**Date and time** (DD/MMM/YY) and (24hrs)

STATEMENT OF PERSON OBTAINING CONSENT

I, the undersigned, certify that to the best of my knowledge the participant signing this consent form had the study fully and carefully explained and clearly understands the nature, risks, and benefits in his/her participation in this research study.

**Name of study team member taking consent** (printed)	**Signature of study team member taking consent**	**Date and time** (DD/MMM/YY) and (24hrs)

Study participant initials/thumbprint: _______________
