# Supplementary material for: Safety and immunogenicity of rVSVΔG-ZEBOV-GP vaccination when dosed concurrent with mRNA COVID-19 vaccine booster doses in healthy African adults (EbolaCov): protocol for a phase IV, single-centre, single-blinded, randomised controlled trial
Source: BMJ Open. 2025 Sep 21;15(9):e102898. doi: 10.1136/bmjopen-2025-102898 (PMC12458633; doi:10.1136/bmjopen-2025-102898)
Supplement: online supplemental file 2 [file bmjopen-15-9-s002.pdf]

EbolaCov: Ubushakashatsi bwo mu cyiciro cya IV bukorerwa mu kigo kimwe gikorerwamo ubushakashatsi, bugamije gusesengura ingaruka n'uko ubudahangarwa bw'umubiri bwitwara nyuma yo guhabwa urukingo rwa rVSVΔG-ZEBOV-GP, igihe rutangiwe rimwe n'urukingo rwo gushimangira rwa mRNA COVID-19 ku bantu bakuru bafite ubuzimana buzira umuze b'abanyafurika.

AGATABO GAKUBIYEMO AMAKURU Y'UBUSHAKASHATSI N'INYANDIKO Y'AMASEZERANO ASOBANUYE

Uratumiwe ngo witabire muri ubu bushakashatsi bugamije gusuzuma ko nta ntangaruka inkingo za Ebola na COVID-19 bitera no kureba uko ubudahangarwa bw'umubiri bwitwara igihe zitangiwe icyarimwe. Ubu bushakashatsi bukorwa na Kaminuza ya Birmingham, Ikigo cy'igihugu gishinzwe ubuzima cy"u Rwanda (RBC) n'ikigo cy'ubushakashatsi ku buzima bw'umuryango.

Mbere yo guhitamo niba ugira uruhare, ni ngombwa kuri wowe gusobanukirwa ibyerekeye ubushakashatsi buvuga n'ingaruka byakugiraho. Turagusaba gufata umwanya wo gusoma amakuru witonze kandi uganire nabandi niba ubishaka. Niba hari ikintu kidasobanutse cyangwa ushaka andi makuru yisumbuyeho, nturagusaba kwegera itsinda rihagarariye ubushakashatsi (ibisobanuro birambuye biri hepfo). Urakoze gufata umwanya wo gutekereza kwitabira mu bushakashatsi.

Ibisobanuro birambuye:

Ikigo cy'ubushakashatsi ku ubuzima bw'umuryango (CFHR) -Projet San Francisco (PSF).
Itsinda rigari ry'ubushakashatsi ku buzima mu Rwanda na Zambiya (RZHRG)
KK19Av 57, agasanduku k'iposita 780, Kigali-Rwanda
www.rzhrg.org

Intego y’ubu bushakashatsi ni iyihe?

Turashaka kureba nib anta ngaruka urukingo Rwa Ebola rutera no kureba uko ubudahangarwa bw’umubiri bwitwara mugihe uru rukingo rutangiwe icyarimwe n’urukingo rwa COVID-19. Urukingo rwa Ebola, rVSVΔG-ZEBOV-GP, rwerekanwe ko ntangaruka rutera kandi ko rubasha kurinda indwara ya virusi ya Ebola (EVD), kandi rwemejwe gukoreshwa mu mwaka wa 2019. Vuba aha, twakoze inkingo nshya zo gukingira indwara ya COVID-19 y’igikatu. Ubu bushakashatsi bwateguwe kugirango harebwe uburyo umubiri witwara myuma yo guhabwa urukingo rwa Ebola mugihe rutangiwe icyarimwe n’urukingo rwa mRNA COVID-19. Tuzareba ko ntanagaruka rutera ndetse n’ingano y’ubwirinzi (abasikare barinda umubiri) kugirango turebe niba ubu buryo bwaduha umurongo w’uko dushobora kugira gahunda nziza zo gukingira mu gihe kizaza.

Incamake y'ubushakashatsi

Dufite intego yo gushyira mu bushakashatsi abantu babishaka 72 bafite ubuzima buzira umuze kandi bafite hagati yimyaka 18 na 50 y’amavuko

Abitabiriye ubushakashatsi ku bushake bazashyirwa mu matsinda mu buryo bwa tombola, bamwe bajye mu itsinda (a) aho bazaterwa urukingo rumwe rwa Ebola mu kuboko kumwe, hamwe n'urukingo rwa COVID-19 inshuro imwe mu kandi kaboko, abanda bajye mu itsinda (b) aho bazatrwa urukingo rwa Ebola inshuro imwe mu kuboko kumwe, n’umuti utagira icyo uvura mu kundi kuboko.

Ntuzigera uhitamo itsinda ry’ubushakashatsi uzashyirwamo, kandi kwitabira ubu bushakashatsi bizamara amezi 6 yose hamwe. Ubushakashatsi niburangira, uzabona kumenya inkingo wahawe ugitangira mu ubushakashatsi , hanyuma uhabwe urukingo rwa COVID-19 niba utari waruhawe mbere.

Buri wese mu bitabiriye tuzamusaba kuzuza inyandiko zerekana ingaruka zose zatewe n’inkingo yahawe yagize mu minsi 7 nyuma yo gukingirwa, kandi azasabwa kuza kuri site y’ubushakashatsi nibura inshuro 4-5 mu gihe cy’amezi 6 kugira ngo abakozi bakora ’ry’ubushakashatsi rishobore gusuzuma imibereho yawe no gukusanya urugero ruto rw’ibizami by’amaraso (mLs 20) rungana na mililitiro 20, kugirango hapimwe ingano y abasirikare barinda umubiri.

Ni izihe nkingo ziri gukoreshwa muri ubu bushakashatsi?

Tuzakoresha inkingo ebyiri zahawe icyemezo cyo gukoreshwa:

- Urukingo rwa Merck Ervebo® Ebola (rVSV-ZEBOV-GP). Uru ni urukingo rurimo virusi nzima rukoresha virusi mwikorezi itwara uturemangingo tw’ imiterere kamere ya virusi ya Ebola kugira ngo umubiri ukore proteyini ya Ebola maze bitere ubudahangarwa bw’ umubiri gukora ubwirinzi bwo kurinda virusi ya Ebola nyirizina mu gihe kizaza . Ntibishoboka kwandura Ebola bitewe n’ urukingo, kandi uru rukingo rwakorewe isuzumwa ryimbitse mu bushakashatsi bwabanje mbere kandi byagaragaye ko ntangaruka rutera kandi rubasha kurinda kandi rwemewe gukoreshwa mu mwaka wa 2019.

- Urukingo rwa Pfizer - BioNTech COVID-19, rugurishwa ku izina rya Comirnaty, ni urukingo rukozwe hashingiyeku miterere kamere ya virusi SARS-CoV-2 rwakoreshejwe neza mu gushyiraho iherezo ry'icyorezo cya COVID-19. Uru rukingo rukoresha uburyo bwo gutanga uturemangingo tw' imiterere kamere kugira ngo umubiri ukore poroteyine ya SARS-CoV-2 maze bitere ubudahangarwa bw'umubiri gukora ubwirinzi bwo kurinda indwara y' igikatu mu gihe kizaza. Ntibishoboka kwandura coronavirus biturutse muri uru rukingo, kandi uru rukingo narwo rwakorewe isuzumwa ryimbitse mu bushakashatsi kandi byagaragaye ko ntangaruka utera kandi rubasha kurinda ndetse rwemejwe gukorehswa mu mpera z'umwaka wa 2020.

Abantu benshi bamaze guhabwa izi nkingo zombi, mubihe bitandukanye mubuzima bwabo, kandi nta mpungenge z'ingaruka zigeze zigaragara.

Ibyibandwaho muri ubu bushakashatsi ni ugusuzuma impinduka izo arizo zose zituruka ku ngaruka / cyangwa uku ubudahangarwa (abasirikare barinda umubiri) bitwara ku rukingo rwa Ebola, mu igihe rutangiwe icyarimwe n'urukingo rwa mRNA COVID-19.

Ni itegeko ko nitabira muri ubu bushakashatsi?

Oya, ibi ni kubushake. Ni wowe ugomba guhitamo niba uzitabira ubushakashatsi . Niba uhisemo kubigiramo uruhare, uzahabwa inyandiko y'amakuru n'amasezerano asobanuye kugirango uyibike (cyangwa uyihabwe mu buryo bw'ikoranabuhanga) hanyuma uzasabwa gushyira umukono ku mpera z'iyi nyandiko y'amasezerano asobanuye. Niba uhisemo kubigiramo uruhare, urashobora guhindura ibitekerezo byawe umwanya uwariwo wose kandi ntukeneye gutanga impamvu iyo ari yo yose.

Ninde ukwiye kwitabira muri ubu bushakashatsi?

Turimo gushaka abantu bashaka kwitabira ubushakashatsi ku bushake bafite ubuzima buzira umuze bafite imyaka 18-50. Byongeye kandi,

- Ugomba kuba witeguye kudusangiza amakuru arebana n'uburwayi waba waragize
- Ugomba kuba ushobora kwitabira randevu zose z'ubushakashatsi inshuro3 mumezi 6
- Ntugomba kuba warigeze kguhabwa urukingo rwa Ebola cyangwa warigeze kwandura virusi ya Ebola
- Ugomba kuba waramaze guhabwa byibuze inshuro ebyiri z'urukingo rwa COVID-19, kandi urwo uheruka guhabwa hagomba kuba harenze amezi 3 uruhawe.
- Ntugomba kuba utwite, kuba wonsa cyangwa uteganya gusama mumezi 6 ari imbere
- Ntugomba kuba ufite ibibazo bikomeye by' ubudahangarwa bw'umubiri wawe, cyangwa kuba ufata imiti iyo ari yo yose igabanya ubudahangarwa bw'umubiri mumezi 6 ashize
- Ntugomba kuba ufite uburwayi bwa kanseri muri kino gihe (keretse kanseri y'uruhu itari mu bwoko bwa melanoma)
- Ntugomba kuba ufite ibibazo bikomeye birebana no kuva amaraso

- Ugomba kwirinda guhura cyane nabantu bafite ubudahangarwa bw'umubiri bufite budakomeye, abana bato (bafite munsi y'umwaka1), n'abagore batwite ndetse / cyangwa bonsa mu gihe cy' ibyumweru 6 nyuma yo gukingirwa
- Ugomba kwirinda guhura n'inyamaswa zororwa mu gihe cy'ibyumweru 6 nyuma yo gukingirwa
- Ntugomba kuba ufite ibibazo byo kuziranirwa n' inkingo izo arizo zose cyangwa proteine z'umuceri
- Ugomba kwemera kudatanga amaraso mubyumweru 6 nyuma y' urukingo
- Ntugomba kuba warafashe izindi nkingo izo arizo zose muminsi 14 mbere yo kwinjira mubushakashatsi
- Ntugomba kuba wagize ibimenyetso by'uwanduye COVID mu minsi 21 ishize.

Bigenda bite nshaka kwitabira ubushakashatsi ?

Turagusa kuvugisha umwe mubagize itsinda rikora ubushakashatsi niba utegaya kwitabira ubushakashatsi. Ubwo uzatumirwa guhura n'umwe mu bagize itsinda rikora ubushakashatsi burambuye kandi asubize ibibazo ibyo aribyo byose ufite. Umaze kugira amakuru yose ukeneye kandi wagize n'igihe cyo gutekereza kwitabira mu bushakashatsi, uzabazwa niba wifuza gukomeza ibindi bikorwa bikurikira harimo gushyira umukono ku nyandiko y'amasezero kandi ukemerera umwe mu bakora ubushakashatsi gusuzuma niba ubikwiriye kwitabira mu bushakashatsi kandi ko nta ngaruka byakugiraho. Ibi byitwa rendevu yo guhitamo abajya mu bushakashatsi kandi aha niho dutangirira gukusanya amakuru akwerekeye. Intego y'iri suzuma rikorwa mu rwego rwo guhitamo abajya mu bushakashatsi ni ukurango hizerwe ko utazagira ibyago byashyira ubuzima bwawe mu kaga.

Ni ayahe makuru ukusanya kuri njye igihe nitabira isuzuma rya mbere (screening)?

- Tukubaza ibibazo bikwerekeye, nk'itariki yawe y'amavuko, inkomoko, amakuru y'uko twakugeraho (aho utuye, terefone na imeri), tukubaza ibibazo bijyanye n'uburwayi waba waragizeaho ibi biri ngombwa kugirango ngo witabire mu bushakashatsi, no kuboneka kwawe ngo, witabira randevu za muganga.
- Dufata ibipima byingenzi by'uko umubiri wawe ukora (uko umutima wawe utera, umuvuduko w'amaraso, uburebure bwawe, ibiro byawe)
- Tuzagusuma umubiri mu buryo busanzwe
- Dufata amaraso ku rutoki hanyuma tugapima virusi itera SIDA
- Dusaba ikizami cy'inkari kugira ngo hapimwe ko udautwite ku bantu b'igitsina bose bafite ubushobozi bwo kubyara

Mu gihe abakora ubashakashatsi bamaze kumenya niba ushobora kwitabira, uzatumirwa kuza kuri rendevu ya mbere (yitwa rendevu ya 1).

Nakwitega iki ndamutse ngize uruhare mu bushakashatsi?

Buri muntu witabiriye ubushakashatsi azatumirwa kwitabira ubushakashatsi inshuro 4 nyuma yo guhitamo abajya mu bushakashatsi, ndetse hazabaho no gukusanya amakuru amwerekeyeho bikorewe aho atuye kandi no gukomeza kuvugana nitsinda ry'abashakashatsi mu gihe cyose cy'ubushakashatsi.

Rendevu ya mbere (cyangwa V1) ni mugihe cyiminsi 5 nyuma ya rendevu ya mbere yo guhitamo abajya mu bushakashatsi.

- Tugenzura ko ugishaka kandi ugishoboye kwitabira mu bushakashatsi
- Dufata mLs 20z'amaraso kugirango dupime urugero rw' abasirikare barinda umubiri mbere yo gukingirwa
- Duhita dukoresha mudasobwa kugirango habeho guhitamo mu buryo bwa tombola itsinda ry'ubushakashatsi ujyamo - yaba wowe cyangwa abashakashatsi ntawe ushobora guhitamo itsinda uhabwa.
- Hanyuma uterwa inshinge ebyiri, rumwe ruhita rutangwa nyuma y'urundi; zombi hakoreshwa urushinge na serenge rumwe ruterwa mu kizigira cy'ukuboko kumwe, urundi rushinge ruterwa mu kizigira cy'ukundi k'ukuboko gutandukanye n'ukwa mbere.
- Mbere yo kugenda, uzategereza iminota 15 nyuma yo gukingirwa kugira ngo ukurikiranwe n'abaganga bashizwe ubushakashatsi
- Ufatwa ko watangiye mu bushakashatsi iyo umaze guhabwa urushinge rwa mbere.
- Uzabasha kumenya ko ko urushing rumwe mu zo uhawe ari doze imwe y'urukingo rwa Ebola - ariko ntuzamenya ukuboko kwateweho iyo doze.
- Uzabasha kumenya ko urushinge rumwe rurimo doze y'urukingo rwa mRNA COVID-19 cyangwa se ari urushinge rutabara (amazi yo mu bwoko bwa saline), ariko ntuzamenya ukuboko yatewemo.
- Uzigishwa ukuntu hakusanywa amakuru mu buryo bw'ikoranabuhanga ndetse hamwe no ku mpapuro, hamwe namakuru arambuye ku bagize itsinda ry'ubushakashatsi, hanyuma uzahabwa igipimo gipima ubushyuhe bw'umubiri n'agacamurongo gapima uburebure uzatahana mu rugo.

Nyuma ya randevu ya mbere uzuzuza inyandiko mu buryo bw'ikoranabuhanga cyangwa ku mpapuro mu minsi 7 ikurikira, gasobanura, buri munsi, ingaruka zose zaturuka ku nkingo. Muri byo harimo;

- Ingaruka z'ahateweho inshinge zombi; harebwe ko ikimenyetso gihari, ubukana bwacyo n'igihe kimara , ububabare, Gutukura k'uruhu, kubyimba kubyimba/gukomera cyangwa mu kubyimba mu kwaha.

- Ingaruka zo mu mubiri; kwandika ubushyuhe bwo mu kanwa no kuboneka, ubukana bwo kubabara umutwe, umunaniro, kubabara imikaya, kubabara mu ngingo , isesemi / kuruka, gutengurwa cyangwa kubabara munda
- Impungenge zose ziyongera kububabare ndetse / cyangwa kubyimba hafi y'ingingo izo arizo zose (harimo hafi y'urwasaya n'imitsi), ibisebe cyangwa ubundi burwayi bw'uruhu (harimo imbere mu kanwa)
- Ibindi bimenyetso cyangwa impungenge

Randevu ya kabiri (cyangwa v2) izaba hagati y'iminsi 7 na 11 nyuma yo gukingirwa

- Tuzareba amakuru wabitse ukoresheshe ikoranabunga cyangwa impapuro niba hari ingaruka zaba zarabaye zitewe n'urukingo, abazaba barujuje amakuru y'ikoranabuhanga bashobora gukoresha telephone n'aho abazaba barabitse amakuru ku mpapuro bagomba kuzagera aho dukorera ubushakashatsi

Gusura kwa gatatu (cyangwa V3) bizaba iminsi 28 (iminsi +/- 4) nyuma yo gukingirwa

- Tugenzura ko ugishaka kandi ugishoboye kwitabira mu bushakashatsi; ibi birimo amakuru mashya yerekeye uko ubuzima bwawe buhagaze
- Dufate ibipima by'ingenzi by'uko umubiri wawe ukora (uko umutima utera , umuvuduko w'amaraso)
- Dufate mL20 y'amaraso kugirango tupime urugero rw'abasirikare barinda umubiri nyuma yo gukingirwa

Gusura kwa kane fourth kandi ari na ko kwa nyuma (cyangwa V4) bizaba amezi 6 (iminsi +/- 14) nyuma yo gukingirwa

- Tugenzura ko ugishaka kandi ushoboye kugira uruhare bushakashatsi; ibi birimo amakuru mashya yerekeye ubuzima bwawe
- Dupima ibimenyetso byingenzi (umuvuduko wumutima, umuvuduko wamaraso)
- Dukusanya 20mLs yamaraso kugirango dupime urugero rwa antibody nyuma yinkingo
- Tukubwira inkingo wahawe ugitangira ubushakashatsi
- Kuri iyi nshuro ugira amahitamo yo guhabwa doze imwe y'urukingo rwa COVID-19 niba utararubonye ugitangira ubushakashatsi .

Ni izihe ngaruka zo wkitabira muri ubu bushakashatsi?

Itsinda ry'abashakashatsi bari hano mbere na mbere hano ku kugirnago umererwe neza. Ingaruka kwitabira mu bushakashatsi zishobora kuvugwa muri make muri ubu buryo;

- Gufata amaraso hakoreshwa urushinge na syringe bishobora gutera ububabare bwigihe gito / kutamererwa neza rimwe na rimwe bigatera impfunira. Dufata amaraso mL20 gusa kuri buri rendevu y' ubushakashatsi (ibi bingana n'utuyiko 5 duto) kandi ibizamini by' amaraso byose bifatwa bingana na mL60 mu mezi 6 y' ubushakashatsi ntacyo byangiza kubuzima bwawe.

- Gukingira bikunze gutera ububabare bw'igihe gito / kutamererwa neza rimwe na rimwe biterwa impfunira aho bateye urushinge. Ibindi bimenyetso biza ahatewe urushinge bishobora kubamo gutukura cyangwa kubyimba kandi wigihe gito cyangwa, kandi ibi bigomba gukira byuzuye mu minsi mike. Rimwe na rimwe, abantu bamwe bumva batameze neza bafite umuriro cyangwa ububabare / uburibwe, ariko ibi biroroshye kandi niby' igihe gito. Inkingo zombi za Ebola na COVID-19 zikoreshwa muri ubu bushakashatsi n' inkingo zemewe, bivuze ko zamaze gukorerwa isuzumwa ryinshi ryimbitse binyuze mu bushakashatsi kandi zemejwe gukoreshwa n'inzego zigenga.

- Ingaruka zikomeye ku rukingo ni ibintu bibaho inshuro nyeya kandi iyo bibaye ibi bikunze kugaragara murwego rwo kuziranirwa. Ingaruka zo kuziranirwa bishyira ubuzima mu kaga ibaho hafi inshuro 1 kuri miliyoni, kandi mu bisanzwe ibi biba nyuma yo guhabwa urukingo mugihe itsinda ry'abashakashatsi rikiri kumwe nawe, kandi barahuguwe gufasha mugihe bikenewe. Urukingo rwa mRNA COVID mu nshuro nke cyane rushobora gutera intege nyeya mu mikaya yo mu maso no / cyangwa no kubyimba umutima (hafi inshuro 1 ku10,000).

Ni izihe nyungu zo kugira uruhare muri uru rubanza?

Inkingo zombi zagaragaje ko zifite ubushobozi mu gukumira indwara. Icyakora, ntituzi niba guhabwa inkingo zombi icyarimwe bihindura ingano y'abasirikare barinda umubiri bakozwe bitewe n'urukingo rumwe cyangwa zombi. Iyi ni imwe mu mpamvu zituma dukora ubu bushakashatsi.

Inyungu zinyongera zirashobora kubamo uburambe bwo kugira uruhare no gutanga umusanzu mubumenyi bwingenzi kubantu bazaza bashobora kungukirwa nakazi dukorana, kandi uzaba wiga kubuzima bwawe bwite.

Ese hari icyo nishyura kubera kwitabira muri ubu bushakashatsi?

Ntacyo usabwa kwishyura kubera ko wemeye kwitabira ubushakashatsi. Uzahabwa 15,000 rwf kuri buri randevu y' ubushakashatsi wakoze kubera igihe cyawe n'amafaranga yose wakoresheje mu kwitabira randevu z' ubushakashatsi.

Ni ibihe bizamini bikorwa ku maraso yanjye?

Ibizami utanga bipimirwa ku kigo ahakorerwa ubushakashatsi i Kigali kandi, niba ubyemera, byapimirwa no muri laboratwali z'abafatanyabikorwa mu Rwanda no mu Bwongereza. Dukora isuzuma ko nta ngaruka kandi dupima ingano y'abasirikari b'umubiri berekana uko ubudahangarwa bw'umubiri

Inyuguti zitangira amazina/ igikumwe cy'uwitabiriye ubushakashatsi:________________

bwitwaye bitewe n' inkingo gusa. Ntabwo dukora ibizamini by'imiterere kamere ibyo aribyo byose, ntitubika cyangwa ngo dukoresha uturemangingo twawe, cyangwa gukora isesengura iryo aririyo ryose usibye gusuzuma ko nta ngaruka zabaye ndetse no gupima abasirikare b'umubiri berekana uko ubudahangarwa bw'umubiri bwitwaye bitewe no gukingirwa.

Ese nzabona uburyo bwo kwivuza bwinyongera kubera kwitabira ubushakashatsi?

Oya. Ntabwo utakaza uburenganzira bwemewe n'amategeko cyangwa ubuvuzi ku bwo kwitabira ubushakashatsi, ariko ntanubwo wongererwa uburenganzira utari usanganwe bwo kwivuza kubera kwitabira ubushakashatsi. Niba ufite ubwishingizi bw'ubuvuzi bwigenga, ushobora gushaka kubaza umwishingizi wawe mbere yo kuwkitabira muri ubu bushakashatsi. Niba utameze neza mugihe cy'ubushakashatsi, itsinda ry'abashakashatsi rizaba rihari kugira ngo bagenzure niba ibi bifitanye isano n'ubushakashatsi. Ukeneye ubuvuzi itsinda ry'abashakashatsi bashobora kubigufashamo.

Byagenda bite niba amakuru mashya abonetse?

Niba amakuru mashya avuye mu bundi bushakashatsi bukoresha inkingo nk'iziri gukoreshwa muri ubu bushakashatsi, amenyeshejwe itsinda rikora ubu bushakashatsi, tuzayakumenyesha kandi tuganire kuri aya makuru ndetse nicyo bishobora gusobanura ku birebana n'uruhare uzaba uri kugira muri ubu bushakashatsi.

Ese nyuma y'ubushakashatsi hari ibindi biganiro bizaba?

Muri bamwe mu bitabiriye ubushakashatsi bazatubwira uko batekereza nuko babonye ubushakashatsi. Kwitabira iki kiganira ni amahitamo, kizakorwa mu buryo bwihariye n'umwe mu bakozi bakora mu bushakashatsi. Ikiganiro kizaba ari ibanga kandi ushobora guhitamo kwitabira ubushakashatsi ariko ugahitamo kutitabira iki kiganiro.

Ni ngombwa kuguma mu bushakashatsi kugeza bushoje?

Oya. Ufite umudendezo wo guhindura imitekerereze yawe no kuva mu bushakashatsi umwanya uwariwo wose. Twakomeza kubika amakuru hamwe n'ibizamini byawe watanze kugeza igihe utubwiye ko wifuza kuva mu bushakashatsi, ariko ntakindi twakubaza keretse niba hari ikibazo kirebana n' ingaruka zitewe n'urukingo. Uruhare rwawe muri ubu bushakashatsi rushobora kandi guhagarikwa igihe icyo aricyo cyose nitsinda ry'abashakashatsi ku bw'impamvu z'ubuzima bwawe.

Ubaye utakiri mu bushakashatsi, igihe cyonyine tuzakomeza kugukurikirana no kuvugana ni igihe tukubaza dukurikirana ku ngaruka runaka wagize uri mu bushakashatsi cyangwa se mu gihe wasamye ku migendekere y'inda wasamye.

Byagenda bite se niba hari ibitagenze neza?

Itsinda ry'abashakashatsi ryemera uruhare rukomeye abitabiriye ubushakashatsi bagira mu bushakashatsi mu rwego bw'ubuvuzi kandi bagakora ibishoboka byose kugira ngo bakurinde ingaruka kandi umerwe neza. Kaminuza ya Birmingham, nk'umuterankunga w'ubushakashatsi, ifite gahunda mu gihe byaba ngombwa ko wagira ingaruka mbi nk'ingaruka zitaziguye zo kwitabira ubu bushakashatsi. Mugihe habaye ibyago bikugarije, mugihe Umuterankunga azaba akurikirana ikibazo icyo aricyo cyose,

ushobora gushaka inama z'amategeko mu buryo bwigenga kugirango wizere ko uhagarariwe neza mugukurikirana ikirego icyo aricyo cyose. Itsinda rihagarariye ubushakashatsi rishobora kukugira inama y'ibindi bikorwa hanyuma bakakohereza kwa muganga nibiba ngombwa.

Ese uruhare rwanjye muri ubu bushakashatsi rwakomeza kugirwa ibanga?

Yego. Kwitabira ubushakashatsi kwawe, amakuru akwerekeyeho hamwe n'ibizami uzatanga byawe byose biguma ari ibanga. Amakuru yose yakusanyirijwe akwerekeyeho yanditseho numero yihariye y'ubushakashatsi ikoreshwa mubyo wanditse byose. Igitabo cy'ukuntu buri kode ihuzwa n'indangamuntu yawe kibikwa neza, ukwacyo kandi ntikigaragazwa hanze mu gihe hari itsinda ryemerewe gukora kuri ubu bushakashatsi. Amakuru yose hamwe n'ibipimo byafaswe kuri wowe bizahita bigirwa ibanga hakoreshejwe umubare wa code uhita uhabwa ukuranga muri ubu bushakashatsi.

Amakuru abikwa mu buryo bwizewe, ahishe neza, kandi uburenganzira bwo kuyageraho bukagirwa n"abagize itsinda ry'ubushakashatsi bafite ikoranabuhanga rigenzura imicungire yizewe y'amakuru. Ikarita y'ikoranabuhanga yoherezwa kuri imeri kugirango amakuru y'ingenzi atazimira kandi aderesi imeri yawe izabikwa kuri seriveri itekanye.

Kugira ngo ubushakashatsi bukorwe neza, abahagarariye abaterankunga b'ubushakashatsi (Kaminuza ya Birmingham) n'inzego zishinzwe kugenzura iby'ubushakashatsi (Ikigo gishinzwe ibiribwa n'imiti mu Rwanda) bashobora gusaba kubona inyandiko zigendanye n'ubu bushakashatsi batabangamiye ibanga ryawe.

Ni iki kizakorwa ku bivuye muri ubu bushakashatsi?

Ibisubizo by'ubu bushakashatsi bizashyirwa ahagaragara mu binyamakuru bya siyansi, ariko ntushobora kumenyekana muri raporo cyangwa igitabo icyo ari cyo cyose. Inyigisho imaze gutangazwa, tuzohereza incamake y'ibisubizo kubantu bose bitabiriye ubushakashatsi. Ntuzigera ubona ibisubizo byawe bwite.

Ninde utera inkunga ubushakashatsi?

Ubushakashatsi bwatewe inkunga na Merck Sharp & Dohme (MSD) na gahunda ya Merck Investigator Studies Program (MISP).

Ninde dushobora kuvugana niba mfite ibibazo, ibitekerezo, ibirego, cyangwa impungenge?

Turizera rwose ko huzagira ibihe byiza byo gukorana nitsinda ry'abashakashatsi. Niba wifuza kuvugana n'umwe muri twe ku bw'impamvu iyo ari yo yose, turagusaba guhamagara umwe mubagize itsinda ry'abashakashatsi bakurikira;

- Umuyobozi mukuru ushinzwe ubushaskashatsi Dr Julien Nyombayire Mutagisha , Ikigo cy'ubushakashatsi ku buzima bw'umuryango, kuri imeri jnyombayire@rzhrg-mail.org cyangwa telephone:+250788308478
- Umushakashatsi wungirije Prof Claude Mambo Muvunyi, Ikigo cy'ubuvuzi cy'u Rwanda, kuri imeri claude.muvunyi@rbc.gov.rw cyangwa telefone +250788493814.

Inyuguti zitangira amazina/ igikumwe cy'uwitabiriye ubushakashatsi:________________

- Umushakashatsi mukuru Dr Christopher Green, kaminuza ya Birmingham, mu Bwongereza, kuri imeri c.a.green.2@bham.ac.uk

Niba ufite ikibazo kijyanye n'uburenganzira bwawe nk'uwitabiriye muri ubu bushakshatsi baza abahagarariye komite y'igihugu ishinzwe kurengera uburenganzira bw'abitabira ubushakashatsi mu Rwanda aribo;

Umuyobozi wa komite ishinzwe kurengera uburenganzira bw'abitabira ubushakashatsi mu Rwanda, Dr. Vedaste Ndahindwa kuri imeri **************** cyangwa telefoni ************.

- Umunyamabanga wa Komite i ishinzwe kurengera uburenganzira bw'abitabira ubushakashatsi mu Rwanda, Dr. Marie Françoise Mukanyangezi kuri emery ****************** cyangwa telefoni **************.

Inyuguti zitangira amazina/ igikumwe cy'uwitabiriye ubushakashatsi:________________

EbolaCov: Ubushakashatsi bwo mu cyiciro cya IV bukorerwa mu kigo kimwe gikorerwamo ubushakashatsi, bugamije gusesengura ingaruka n'uko ubudahangarwa bw'umubiri bwitwara nyuma yo guhabwa urukingo rwa rVSVΔG-ZEBOV-GP, igihe rutangiwe rimwe n'urukingo rwo gushimangira rwa mRNA COVID-19 ku bantu bakuru bafite ubuzimana buzira umuze b'abanyafurika.

URUPAPURO RW' IMIKONO KU NYANDIKO Y'AMASEZERANO ASOBANUYE

Nemeye ibi bikurikira:	Shyira inyuguti zitangira amazina yawe / igikumwe muri buri kazu
Ndemeza ko nasomye kandi numvise iyi nyandiko y'amakuru y'ubushakashatsi hamwe n'amasezerano asobanuye yo kwitabira mu bushakashatsi. Nagize amahirwe yo gusuzuma amakuru, kubaza ibibazo, kandi nasubijwe ibyo bibazo ndanyurwa.	
Ndumva neza ko kwitabira ubushakashatsi ari ubushake bwanjye kandi mfite umudendezo wo kubivamo igihe icyo ari cyo cyose ntatanze impamvu, ntacyo bihindura ku burenganzira bwanjye ku buvuzi cyangwa mu mategeko.	
Ndumva neza ko ikusanyamakuru mugihe cy'ubushakashatsi, harimo namakuru yanjye yubuvuzi, rishobora kurebwa n'abantu baturutse ku muterankunga, uhagarariye ubushakashatsi hamwe na/cyangwa abayobozi bakurikirana iby'ubushakashtsi, aho ari ngombwa ko ngira uruhare muri ubu bushakashatsi. Ndatanga uburenganzira kuri abo bavuzwe haruguru kugirango babone inyandiko zanjye.	

Inyuguti zitangira amazina/ igikumwe cy'uwitabiriye ubushakashatsi:_______________

Ndumva ko amakuru yanjye bwite, yaba n'ahishwe mu buryo bwa kode (code) azakomeza gutunganywa nyuma y'uko ubushakashatsi burangiye cyangwa nyuma yo kuva mubushakashatsi butarangiye bibaye ngombwa, ku impamvu z'inyungu rusange m'ubuzima rusange, kubikorwa byo kubika inyandiko ku nyungu rusange, kubikorwa byubushakashatsi bwa siyansi, cyangwa kubikorwa n'ibarurishamibare.	
Ndumva neza ko amakuru yakusanyijwe anyerekeyeho azakoreshwa mugushyigikira ubundi bushakashatsi mugihe kizaza kandi ashobora gusangizwa n'abandi bashakashatsi mu buryo hatamenyekana uwo ndiwe..	
Nemeye ibyerekeye ikoreshwa ry'ibizamini nzatanga ku mpamvu zasobanuwe muri iyi nyandiko y'amakuru n'amasezerano asobanuye.	
Ndumva neza ko nzahabwa kopi y'iyi nyandiko y'amakuru n'masezerano asobanuye iriho imikono,. Niba nanze gufata kopi y'iyi nyandiko rwabigenewe, ushinzwe ubushakashatsi ashobora kuyimbikira ahizewe hubahirijwe ibanga ku kigo gikorerwaho ubushakashatsi ..	
Nemeye kwitabira muri ubu bushakashatsi ku bushake bwanjye bwite.	
[**Ibi si ngombwa cyane**] Ndemera ko ibizamini by'amaraso yose asigaye muri ubu bushakashatsi ku byo nzatanga ashobora gukoreshwa mu bushakashatsi bujyanye n'urukingo n'ubundi bushakashatsi ku budahangarwa. Ibi bishobora kuba bikubiyemo kohereza ibizami muri laboratwari mu bigo bifitanye ububanyi n'Ikigo cy'Ubuzima mu Rwanda (RBC) cyangwa mu Bwongereza (United Kingdom : University of Birmingham). Ndumva ko umwirondoro wanjye uzakomeza kuba ibanga, ko ndamutse nanze iki cyemezo ku bushake nshobora gukomeza kwitabira mu bushakashatsi , kandi ubwo bushakashatsi bwo mu gihe kizaza bwakorwa ari uko bwemejwe n'abakurikirana ubushakashatsi	

**Izina ry'uwitabiriye ubushakashatsi**<br>(mu cyapa)	**Umukono/igikumwe cy'uwitabiriye ubushakashatsi**	**Itariki n'isaha**<br>(DD/MMM/YY) and<br>(24hrs

Inyuguti zitangira amazina/ igikumwe cy'uwitabiriye ubushakashatsi:________________

Ibyo Umutangabuhamya udafite aho abogamiye yemeza (Niba uwitabiriye ubushakashatsi atazi gusoma no kwandika):

Nshyize umukono hano nk'umutangabuhamya w'uko amasezerano yasobanuwe. Nakurikiye ibyaganiriweho kandi ndemeza ko uwo mbereye umutangabuhamya yemeye ku bushake bwe kujya mu bushakashatsi

**Izina ry'umutangabuhamya wigenda(mu cyapa)**	**Umukono**	

Ibyo umukozi ukora mu bushakashatsi wakiriye amasezerano yemeza

Njyewe, ushyizeho umukono, ndemeza ko nkurikije uko mbibona, uwitabiriye ubushakashatsi ushyize umukono kuri iyi nyandiko y'amasezerano, yasobanuriwe neza mu buryo bwitondewe kandi yumvise neza uko ubushakashatsi buteye, ingaruka, n'inyungu zo kwitabira muri ubu bushakashatsi.

**Izina ry'umukozi uri mu itsinda rikusanya amakuru mu bushakashatsi** (mu cyapa)	**Umukono**	**Itariki n'isaha** (DD/MMM/YY) and (24hrs)

Inyuguti zitangira amazina/ igikumwe cy'uwitabiriye ubushakashatsi:______________
