# Supplementary material for: Safety and immunogenicity of rVSVΔG-ZEBOV-GP vaccination when dosed concurrent with mRNA COVID-19 vaccine booster doses in healthy African adults (EbolaCov): protocol for a phase IV, single-centre, single-blinded, randomised controlled trial
Source: BMJ Open. 2025 Sep 21;15(9):e102898. doi: 10.1136/bmjopen-2025-102898 (PMC12458633; doi:10.1136/bmjopen-2025-102898)
Supplement: Supplementary file 3 [file bmjopen-2025-102898.reviewer_comments.pdf]

PEER REVIEW HISTORY

BMJ Open publishes all reviews undertaken for accepted manuscripts. Reviewers are asked to complete a checklist review form and are provided with free text boxes to elaborate on their assessment. These free text comments are reproduced below.

ARTICLE DETAILS

Title (Provisional)

Safety and immunogenicity of rVSVΔG-ZEBOV-GP vaccination when dosed concurrent with mRNA COVID-19 vaccine booster doses in healthy African adults (EbolaCov): protocol for a phase IV, single-centre, single-blinded, randomised controlled trial

Authors

Gokani, Karishma; Taylor, Alice; Packham, Alice; Musabyimana, Jean Pierre; Shema, Hugor; Mutabaruka, Ariane; Roche, Siobhan; Takwoingi, Yemisi; Umuhoza, Claudine; Nyombayire, J; Muvunyi, Claude; Green, Christopher

---

VERSION 1 - REVIEW

**Reviewer**	**1**
**Name**	**Babiuk, Shawn**
**Affiliation**	**University of Manitoba**
**Date**	**13-Jun-2025**
**COI**	**None**

The authors primary aim of is to determine if the safety and immunogenicity of the rVSVΔG-ZEBOV-GP vaccine with a concurrent SARS-CoV-2 mRNA vaccine using a single-centre, randomized, single-blinded, vaccine safety and immunogenicity study in healthy adults living in Rwanda. Antibody responses will be assessed at 28-days and 180-days after vaccination using a GP-ELISA.

This is important work to determine if the rVSVΔG-ZEBOV-GP vaccine can be used together with a mRNA vaccine.

Reviewer **2**

Name **Choi, Edward**

Affiliation **London School of Hygiene & Tropical Medicine, Department of Clinical Research**

Date **21-Jul-2025**

COI None

Line 102-103

Can the authors please clarify what the following sentence means?

The mRNA COVID-19 vaccines manufactured by Moderna and Pfizer have been principally used in the vaccination effort against SARS-CoV-2.

Line 105-106

Can the authors please clarify the following sentence? Did 21,720 participants received the vaccine in one study or two studies? Please kindly add the relevant citations.

Clinical trials of Comirnaty also reported a good safety profile in two clinical studies where 21,720 participants received the vaccine.

Line 178-181

Can the authors please clarify if the secondary objective will assess the non-inferior of antibody responses to SARS-CoV-2 Spike, when the two vaccines are co-administered, compared to BioNTech – Pfizer COVID-19 mRNA vaccine booster alone?

Secondary objectives include longitudinal assessment of humoral response to rVSVΔG-ZEBOV-GP and SARS-CoV2 when co-administered with BioNTech – Pfizer COVID-19 booster vaccination, compared to when rVSVΔG-ZEBOV-GP vaccine is given alone, and assessment of SARS-CoV-2 anti-Spike protein specific geometric mean titres (GMT) when co-administered with rVSVΔG-ZEBOV-GP vaccine.

Line 209-216

Group 1 participants will receive the COVID-19 mRNA vaccine in the same arm as the Ebola VSV vaccine, albeit with a 6 months delay. Group 2 participants will receive the COVID-19 mRNA vaccine in the opposite arm to the Ebola VSV vaccine in the same visit. There are conflicting evidence as to whether immunizing the same or opposite arm can influence the peak response or kinetics of vaccines and boosters. How will the authors control for any potential effect of immunizing the same vs different arms, in order to conclude that any difference observed in the EBOV GP-antibody titre between Group 1 and 2 is due to co-administration with the COVID-19 mRNA vaccine?

Page 14, Table 1, Line 20

20mLs

Typo. Symbols for International System of Units should never be pluralized. i.e. 20 mL, not 20 mLs.

VERSION 1 - AUTHOR RESPONSE

Reviewer 1 comments
Thank you for your comments. We agree this is important work and one which may gain added significance in the future and the development of other mRNA vaccine products.

Reviewer 2 comments
Thank you for your comments and suggestions, which we have addressed below;
1. Line 102-103
Can the authors please clarify what the following sentence means?
The mRNA COVID-19 vaccines manufactured by Moderna and Pfizer have been principally used in the vaccination effort against SARS-CoV-2.
a. The majority of vaccine doses used in the vaccination effort against SARS-CoV-2 were produced by these two manufacturers. This has been updated in the manuscript with a reference for clarity.

2. Line 105-106
Can the authors please clarify the following sentence? Did 21,720 participants receive the vaccine in one study or two studies? Please kindly add the relevant citations.
Clinical trials of Comirnaty also reported a good safety profile in two clinical studies where 21,720 participants received the vaccine.
a. Line 105-107 were referring to the same single study. This has now been correctly referenced and rewritten for clarity.

3. Line 178-181
Can the authors please clarify if the secondary objective will assess the non-inferior of antibody responses to SARS-CoV-2 Spike, when the two vaccines are co-administered, compared to BioNTech – Pfizer COVID-19 mRNA vaccine booster alone?

Secondary objectives include longitudinal assessment of humoral response to rVSVΔG-ZEBOV-GP and SARS-CoV2 when co-administered with BioNTech – Pfizer COVID-19 booster vaccination, compared to when rVSVΔG-ZEBOV-GP vaccine is given alone, and assessment of SARS-CoV-2 anti-Spike protein specific geometric mean titres (GMT) when co-administered with rVSVΔG-ZEBOV-GP vaccine.
a. The study is not powered for the detection of non-inferior antibody responses to the Spike protein of SARS-CoV-2. We have addressed this in the 'sample size calculation' section. The wide distribution of anti-Spike IgG responses results in an unpracticable sample size of 359 participants to possibly achieve the statistical power to answer this. We will, however, publish all immunology data and intend to undertake post-doc analyses.

4. Line 209-216
Group 1 participants will receive the COVID-19 mRNA vaccine in the same arm as the Ebola VSV vaccine, albeit with a 6 months delay. Group 2 participants will receive the COVID-19 mRNA vaccine in the opposite arm to the Ebola VSV vaccine in the same visit. There are conflicting evidence as to whether immunizing the same or opposite arm can influence the peak response or kinetics of vaccines and boosters. How will the authors control for any potential effect of immunizing the same vs different arms, in order to conclude that any difference observed in the EBOV GP-antibody titre between Group 1 and 2 is due to co-administration with the COVID-19 mRNA vaccine?
a. The primary immunological objective of the study concerns the antibody response to rVSVΔG-ZEBOV-GP vaccine, which will be administered to all participants in the non-dominant arm at the start of the study only. The COVID-19 mRNA vaccine will be administered to group 2 on the last visit following the final visit without further post-vaccination sampling. Any change in which arm is used to administer the COVID-19 mRNA vaccine at the end of the study for those who wish to receive this will have not be recorded or have influence on the primary immunological outcome measures and study validity.

5. Page 14, Table 1, Line 20
20mLs
Typo. Symbols for International System of Units should never be pluralized. i.e. 20 mL, not 20 mLs.
a. This has been corrected.

We remain at your disposal for any further corrections or comments and I look forward to hearing from you soon.

VERSION 2 - REVIEW

Reviewer 2

Name Choi, Edward

Affiliation London School of Hygiene & Tropical Medicine, Department of Clinical Research

Date 02-Sep-2025

COI

No further comments.
